# Supplementary material for: Observation of a non-reciprocal skyrmion Hall effect of hybrid chiral skyrmion tubes in synthetic antiferromagnetic multilayers
Source: Nat Commun. 2025 Sep 26;16:8285. doi: 10.1038/s41467-025-63759-7 (PMC12474975; doi:10.1038/s41467-025-63759-7)
Supplement: Supplementary file 1 — Supplementary Information [file 41467_2025_63759_MOESM1_ESM.pdf]

Observation of a non-reciprocal skyrmion Hall effect of hybrid chiral skyrmion tubes in synthetic antiferromagnetic multilayers

Takaaki Dohi[1, 2*†], Mona Bhukta[2†], Fabian Kammerbauer[2†], Venkata Krishna Bharadwaj[2], Ricardo Zarzuela[2*], Aakanksha Sud[1, 3], Maria-Andromachi Syskaki[2, 4], Duc Minh Tran[2], Thibaud Denneulin[5], Sebastian Wintz[6, 7], Markus Weigand[6, 7], Simone Finizio[8], Jörg Raabe[8], Robert Frömter[2], Rafal E Dunin-Borkowski[5], Jairo Sinova[2] and Mathias Kläui[2, 9*]

[1] Laboratory for Nanoelectronics and Spintronics, Research Institute of Electrical Communication, Tohoku University, Sendai 980-8577, Japan.

[2] Institut für Physik, Johannes Gutenberg-Universität Mainz, Staudingerweg 7, 55128 Mainz, Germany.

[3] Frontier Research Institute for Interdisciplinary Sciences, Tohoku University, Sendai 980-0845, Japan.

[4] Singulus Technologies AG, 63796 Kahl am Main, Germany.

[5] Ernst Ruska-Centre for Microscopy and Spectroscopy with Electrons, Forschungszentrum Jülich, 52425 Jülich, Germany.

[6] Max Planck Institute for Intelligent Systems, Heisenbergstrasse 3, D-70569 Stuttgart, Germany.

[7] Helmholtz-Zentrum Berlin für Materialien und Energie GmbH, Hahn-Meitner-Platz 1, Berlin, D-14109, Germany.

[8] Swiss Light Source, Paul Scherrer Institut, Forschungsstrasse 111, 5232 Villigen PSI, Switzerland.

[9] Center for Quantum Spintronics, Norwegian University of Science and Technology, 7491 Trondheim, Norway.

[†]**These authors contributed equally to this work.**

***Corresponding author(s). E-mail(s):**

Dr. Takaaki Dohi: takaaki.dohi.e5@tohoku.ac.jp

Dr. Ricardo Zarzuela: rzarzuela@uni-mainz.de

Prof. Dr. Mathias Kläui: klaeui@uni-mainz.de

SUPPLEMENTARY INFORMATION

Note 1. Antiferromagnetic coupling before and after current pulsing

Note 2. Lorentz transmission electron microscopy imaging

Note 3. Ferromagnetic resonance for the damping, effective spin Hall angle, etc.

Note 4. Analytical formula for the skyrmion Hall angle

Note 5. Micromagnetic modelling

Note 6. Systematic micromagnetic simulation: magnetic compensation dependence of the static and dynamic properties

Note 1
Antiferromagnetic coupling before and after current pulsing

Decoupling the antiferromagnetically coupled skyrmion tubes in the synthetic antiferromagnetic (SyAFM) system (splitting one SyAFM skyrmion into two ferromagnetic skyrmions or irreversible antiferromagnetic-ferromagnetic transition of the SyAFM system by heating) significantly modifies the current-induced dynamics, including the skyrmion Hall effect[1-3]. To investigate the coupling state of SyAFM skyrmion tubes before and after current pulsing, we observed the SyAFM skyrmion tubes at the L_3-edge of both Fe and Co using the scanning transmission X-ray microscopy (STXM) after multiple pulses. As shown in supplementary Fig. 1, we have unambiguously observed a stable antiferromagnetic coupling of SyAFM skyrmion tubes before and after multiple current pulses (five times), indicating that the strength of interlayer exchange coupling is sufficiently large to overcome the opposite Magnus force at the two sub-lattices and retain their antiferromagnetic (AFM) coupling before and after the motion[3]. Thus, the decoupling does not occur before and after the current-induced motion in the range of current density and magnetic fields used in this work. Note that although some skyrmion tubes could be annihilated owing to heating, all available skyrmion tubes are fully antiferromagnetically coupled, as seen in supplementary Fig. 1.

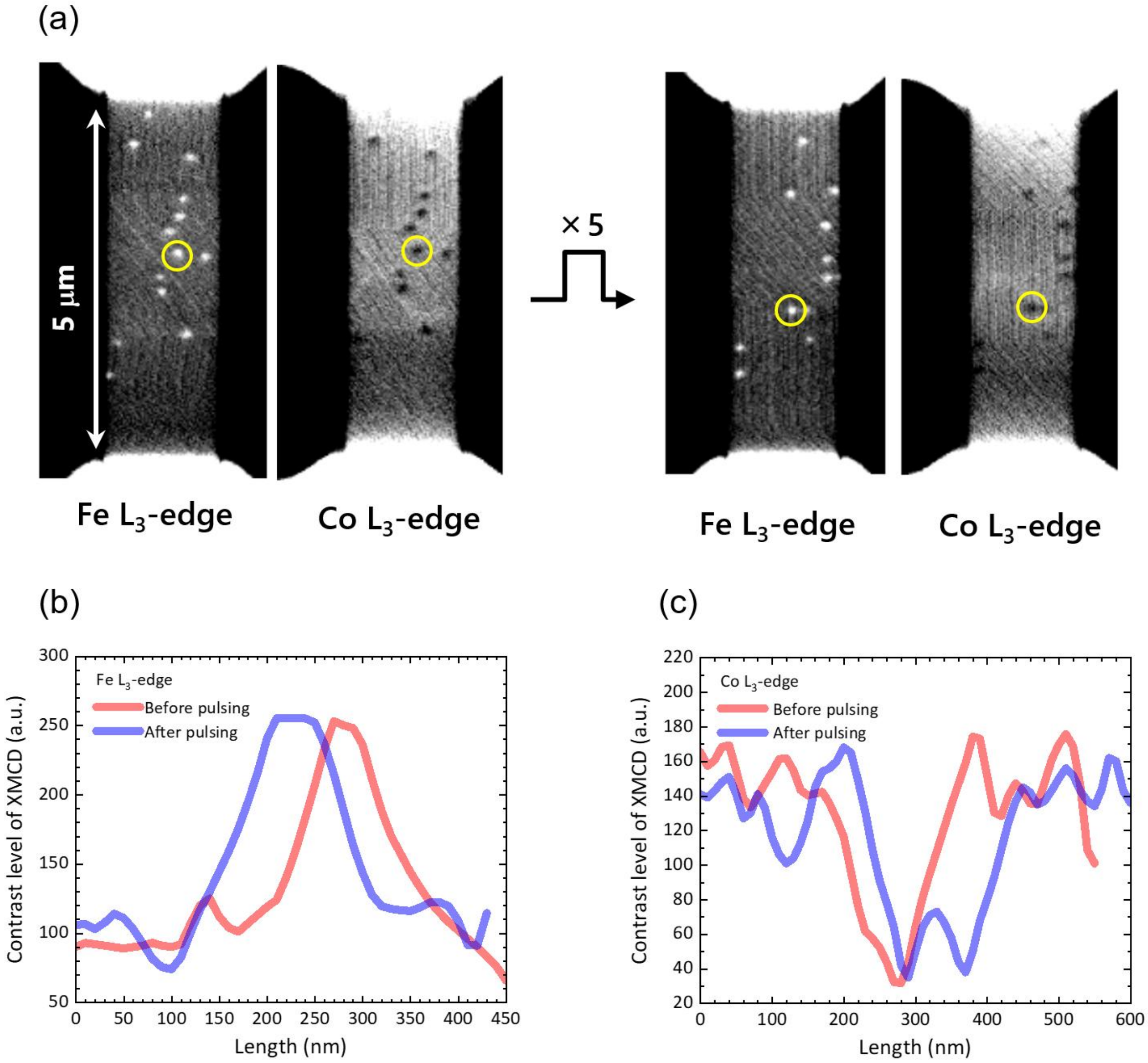

Supplementary Figure 1 | Direct observation of stable antiferromagnetic coupling of skyrmion tubes. **a,** the stack ST2 before (left Fig.) and after (right Fig.) five times the current pulses. The current density of the injected pulse was $J = 9.7\times10^{11}$ A m^{-2}. A perpendicular magnetic field of $\mu_0 H_z$ = 130 mT was applied at room temperature. Panels b and c denote exemplary contrast profiles along the wire-edge direction for each skyrmion highlighted in yellow in Fig.1a. **b**, Fe L_3-edge contrast and **c**, Co L_3-edge contrast. Red and blue curves denote measurements taken before and after current pulsing, respectively.

Note 2
Lorentz transmission electron microscopy imaging

Here, we provide direct evidence of the presence of the Bloch components of domain walls in the ST1 stack to substantiate our discussion and explanations in the main text. The Lorentz transmission electron microscopy (TEM) imaging was carried out on a 50 % compensated SAF deposited onto a SiN membrane using a TFS Titan transmission electron microscope operated at 300 kV in Lorentz mode. The objective lens was used to apply a magnetic field along the electron beam direction that was pre-calibrated using a Hall sensor. Images were acquired using a Gatan UltraScan 1000XP CCD camera.

Supplementary Figure 2a and b are Lorentz TEM images obtained at a tilt angle of 0° and 10°, respectively, that show stripe domains. In out-of-plane magnetized samples, the contrast at 0° depends on the type of domain wall. Bloch domain walls produce contrast, and the Néel-type do not. In a tilted condition, the contrast depends on both the domain wall type and the projection of the out-of-plane component of the magnetic field in the image plane[4]. Here, the magnetic contrast is particularly weak at 0° compared to 10°, which indicates that the magnetic texture is essentially Néel-type. To obtain more information, profiles were extracted from the images across domains as shown in supplementary Fig. 2c and d. In supplementary Fig. 2c, the profiles show a bright/dark/bright distribution as indicated by three arrows in the plots. This distribution indicates the presence of a small Bloch component[5]. In supplementary Fig. 2d, the blue profile was extracted from a domain oriented perpendicular to the tilt axis, which shows a strong dark/bright distribution, as expected from the projection of the out-of-plane component. The red profile in supplementary Fig. 2d was extracted from a domain oriented parallel to the tilt axis. It shows again a weak bright/dark/bright distribution similar to that obtained at 0°. This is another indication of the presence of a small Bloch component. At a tilt angle of 10°,

the projection of the out-of-plane magnetization along the imaging direction is approximately sin 10° ≈ 17 %. Given that the Bloch component contributes to only one-sixth of the full contrast under such geometry, its relative contribution is estimated to be ~3 %.

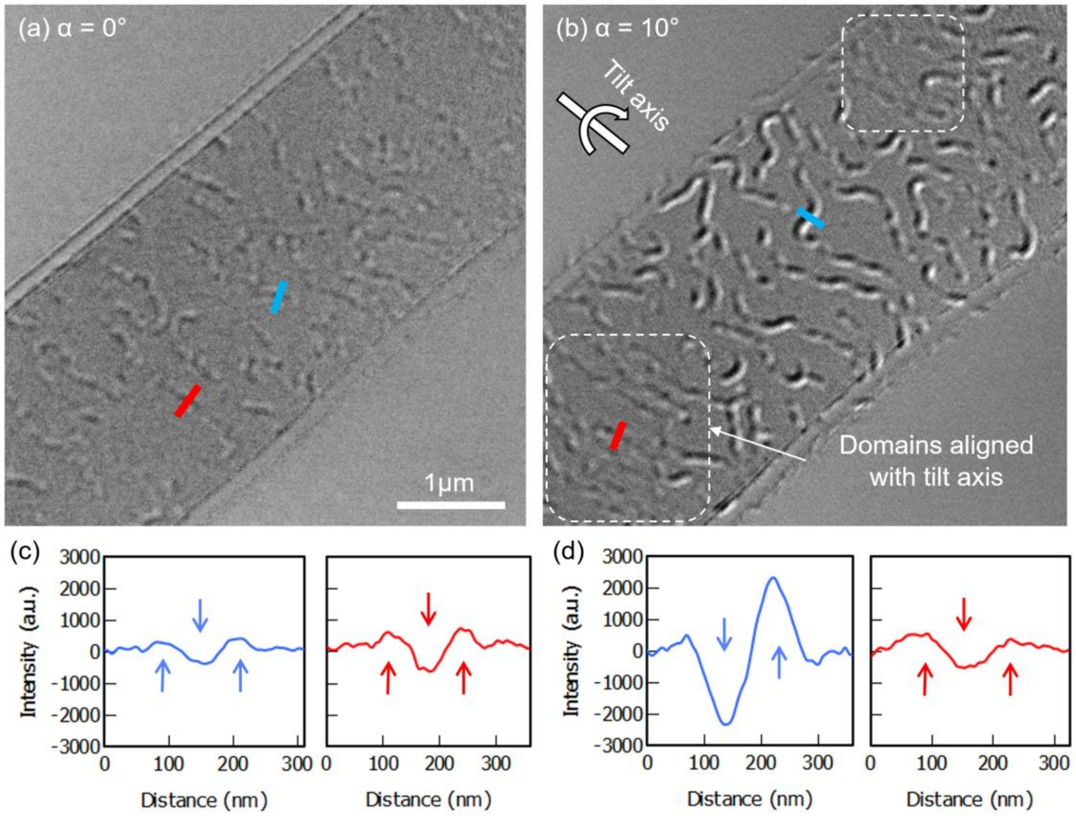

Supplementary Figure 2 | Lorentz TEM images obtained with a defocus of 4 mm, an external magnetic field of 85 mT, and a tilt angle of **a,** α = 0°, and **b**, α = 10°. The non-magnetic background was subtracted using images acquired after saturation[6]. Upon tilting, the domain contrast is enhanced for those aligned along the tilt axis, indicating the presence of in-plane magnetization components. **c,** and **d,** line profiles extracted from the blue and red lines in panels **a** and **b**, respectively. The polarity and asymmetry in the contrast across individual domain walls show the mixed Néel-Bloch character, consistent with hybrid skyrmions that lend themselves to the observed effects.

Note 3

Ferromagnetic resonance for the damping, effective spin Hall angle, etc.

The sample properties, such as the effective damping parameter and the interlayer exchange coupling field $\mu_0 H_{ex}$ for the SyAFM systems, were obtained using the technique of ferromagnetic resonance spectroscopy[7,8]. The sample (ST1 in the main text) was placed upside down on a co-planar waveguide, and broadband spin dynamics characterization was done using a technique similar to Ref. 7,8. By sweeping the external magnetic field, which was modulated with a small AC component of a few Oersteds, we obtained the resonance spectra for different microwave frequencies. Supplementary Figure 3a shows the color plot and the resonance frequencies obtained by fitting a sum of symmetric and anti-symmetric Lorentzian[9]. This protocol is carried out in the case of the field applied in the direction perpendicular to the microwave RF field. The measurements were repeated for the field applied parallel to the RF field. (see blue dots in supplementary Fig. 3b. The resonance frequencies were fitted using Eqs. (1) and (2) given in Ref. 7. We extracted the values of quadratic, $\mu_0 H_{ex}$, and biquadratic exchange field, $\mu_0 H_{bi\text{-}ex}$, as 0.15±0.01 T and 0.018±0.002 T, respectively. The linewidth was also extracted from the fitting of the resonance spectra by using a theoretical model in Ref. 7 and is shown in supplementary Fig. 3c. The solid lines in supplementary Fig. 3c are the fitting curves from which we obtain the effective damping parameter α = 0.1±0.01. This large phenomenological damping possibly stems from the multiple ultra-thin Ta layers inserted between the CoB to reduce pinning effects.

We also quantified the spin-orbit parameters through a series of spin torque-ferromagnetic resonance (ST-FMR) measurements following the technique used in Ref. 10. A typical resonance curve obtained for the acoustic mode is shown in supplementary Fig. 4a which was fitted to obtain symmetric and anti-symmetric components of voltage denoted as V_{sym} and V_{asym},

respectively. From the angular dependence of V_{sym} and V_{asym}, we can quantify the spin-torque parameters. We note that experimentally we could only detect the acoustic mode from ST-FMR measurements and so we present data only for this mode. However, the parameters extracted from optical mode data should be similar as already shown in previous work[10]. For the measurements, a thin bar of 5 μm-width and 40 μm-length was fabricated by standard lithography technique (the sample ST1 in the main text), and a Cr/Au waveguide was deposited on top through which we injected the microwave current at a fixed frequency of 5 GHz while sweeping the magnetic field in different in-plane directions with respect to the bar. The definition of the azimuthal angle θ is shown in supplementary Fig. 4. The ST-FMR spectra were recorded for each angle and fitted using Eq. (3) in Ref. 10. The V_{sym} and V_{asym} components obtained from the fitting are plotted in supplementary Fig. 4c and 4d, respectively, and it can be seen that both components exhibit a $\sin 2\theta \sin\theta$ dependence as expected in Eqs. (S1) and (S2). The resultant torque parameters were quantified by fitting the data in supplementary Fig. 4c and 4d to Eqs. (S1) and (S2) obtained from Ref. 10. The parameters obtained are summarised in supplementary Table 1.

$$V_{\mathrm{sym}} = \frac{-\Delta R_{\mathrm{AMR}}}{8\Delta B_{\mathrm{ac}}\sqrt{1+M_{\mathrm{S}}/(2B_{\mathrm{ex}})}} I_0 B_{\mathrm{SHE}} \sin\theta_{\mathrm{c}} \cos 2\theta_{\mathrm{c}} \sin 2\theta \sin\theta \qquad \text{(S1)}$$

$$V_{\mathrm{asym}} = \frac{\Delta R_{\mathrm{AMR}}}{8\Delta B_{\mathrm{ac}}} I_0 B_{\mathrm{Oe}} \tan\theta_{\mathrm{c}} \cos 2\theta_{\mathrm{c}} \sin 2\theta \sin\theta \qquad \text{(S2)}$$

The details of the theoretical equations can be followed up from Ref. 10. In Eqs. (S1) and (S2), the B_{Oe} is the Oersted field, and B_{SHE} is the spin Hall field which is given as $B_{\mathrm{SHE}} = \frac{\mu_0 \hbar \eta_{\mathrm{HM}} \theta_{\mathrm{SH}} I_0}{2e M_{\mathrm{S}} w d_{\mathrm{FM}} d_{\mathrm{HM}}}$, and $B_{\mathrm{Oe}} = \frac{\mu_0 \eta_{\mathrm{asym}} I_0}{2w}$ and the terms $\hbar$, η_{HM}, I_0, e, w, d_{FM}, d_{HM}, ΔR_{AMR}, θ_{c}, ΔB_{ac}, M_{S}, B_{ex}, η_{asym}, and θ_{SH} refer to the Dirac constant, the shunt ratio of current in the heavy metal layer, the current amplitude in the device, elementary charge, the width of the microbar, the thickness of the total ferromagnetic (FM) and heavy metal (HM) layers, the anisotropic magnetoresistance (AMR)

change, the canting angle, the linewidth, the saturation magnetization, the exchange field, an asymmetry factor of electric currents between the HM and FM layers, and the spin hall angle. The ratio of $B_{SHE}/B_{Oe} = -6.8$, indicates that the field-like component is smaller than the damping-like torque contribution, which mainly drives the skyrmion tube as described in the main text.

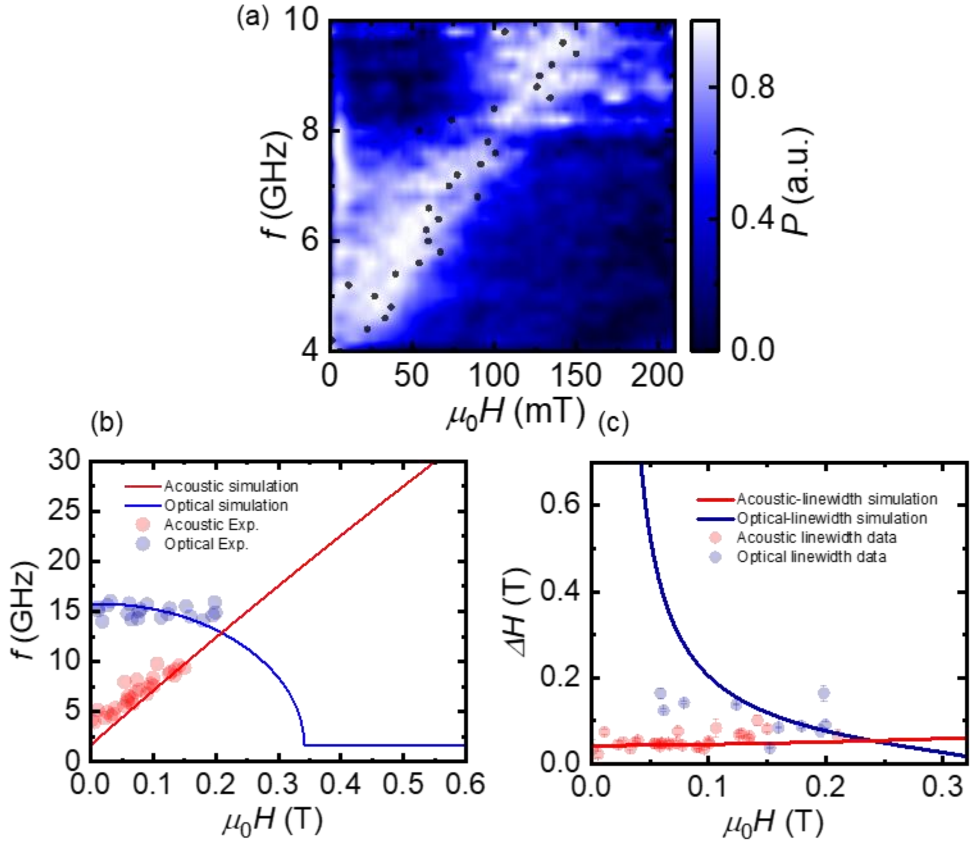

Supplementary Figure 3 | Ferromagnetic resonance characterizing the magnetic properties. **a,** Resonance spectra for the field applied parallel to the injected microwave current. **b**, Resonance frequency as a function of the field for two modes obtained by fitting the resonance spectra. The solid lines are the fittings. **c**, The resonance linewidth extracted for the spectra in supplementary Fig. 3a.

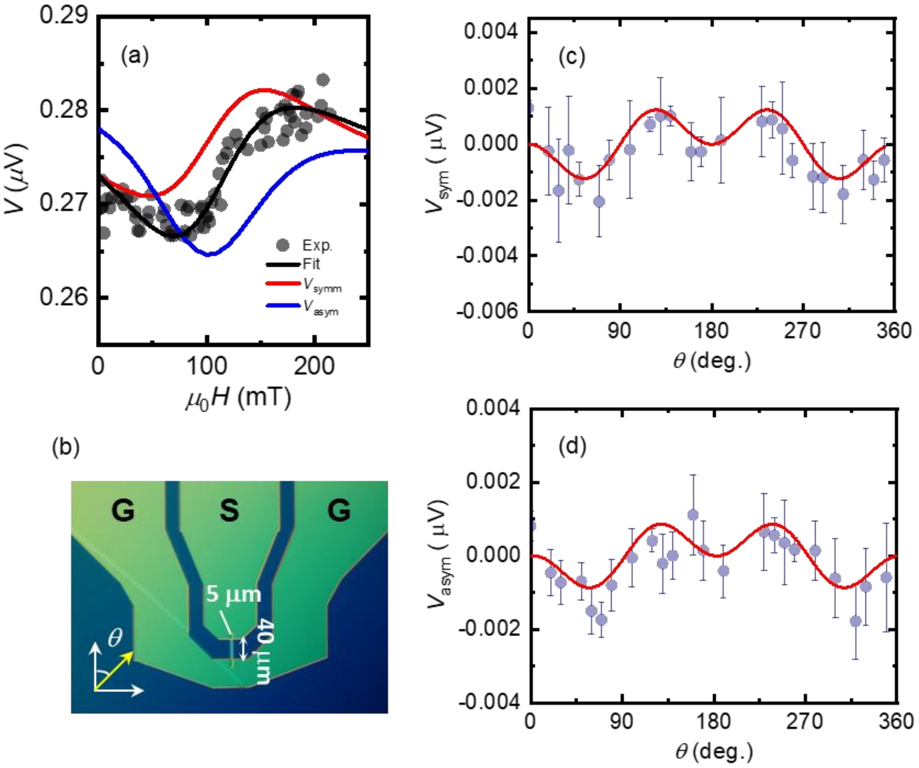

Supplementary Figure 4 | Spin torque-ferromagnetic resonance (ST-FMR) characterization. **a,** Microwave absorption spectra for the acoustic mode at 5 GHz resonance frequency and $\theta = 40$ deg. **b,** Microscope image of the device used. **c,** V_{sym}, and **d,** V_{asym} components as a function of θ. The solid lines are the fittings obtained using Eqs. S1 and S2.

Supplementary Table 1 | The ST-FMR-related parameter list was obtained experimentally and used for the micromagnetic simulations of the current-induced dynamics of the SyAFM skyrmion tube.

Parameters	Magnitudes
Spin Hall field, B_{SHE}	-43 ± 1.5 μT
Oersted field, B_{Oe}	6.3 ± 0.2 μT
Linewidth, ΔB_{ac}	0.15 ± 0.03 T
Current amplitude, I_0	10 ± 0.5 mA
Width of the microbar, w	5 μm
Saturation magnetization, M_{s}	0.9 T
AMR change, ΔR_{AMR}	7 ± 1 mΩ
Asymmetry factor, η_{asy}	5 ± 0.2 ($\times10^{-3}$)
Exchange field, B_{ex}	0.2 ± 0.04 T
Shunt ratio of current, η_{HM}	0.65 ± 0.1
Spin hall angle, θ_{SH}	-0.1 ± 0.05

Note 4

Analytical formula for the skyrmion Hall angle

In this section, we present our analytical treatment of the skyrmion dynamics in a synthetic antiferromagnetic (SyAFM) system. We model the SyAFM system as a multilayer stack of ferromagnetic layers separated by a metallic spacer, which are coupled antiferromagnetically through interlayer exchange interactions. The energy functional includes the key interactions required to stabilize magnetic skyrmions: ferromagnetic exchange interactions within each layer, Dzyaloshinskii-Moriya (DM) interactions, and on-site uniaxial anisotropy. Consequently, the energy functional for each layer, i, describing the multilayer SyAFM reads:

$$\mathcal{E}[\mathbf{m}_i] = \int_S d^2r \left\{A_\mathrm{S}(\nabla\mathbf{m}_i)^2 + D\big[\mathbf{m}_i \cdot \big(\widetilde{\nabla} \times \mathbf{m}_i\big)\big] - K_{\mathrm{eff},i} m_{i,z}^2 - \delta_\mathrm{ex}\big(\mathbf{m}_i \cdot \mathbf{m}_j\big)\right\}, \quad \text{(S3)}$$

where $D\big[\mathbf{m}_i \cdot \big(\widetilde{\nabla} \times \mathbf{m}_i\big)\big] = D\big[(\mathbf{m}_i \cdot \nabla) m_{z,i} - m_{z,i}(\nabla \cdot \mathbf{m}_i)\big]$ and $\mathbf{m}_i(r)$ denotes the order parameter of the i_th-layer, normalized by the saturation magnetization $M_{\mathrm{S},i}$. Here, A_S and D are the spin stiffness constant and the DM strength of the FM layers, respectively, and δ_ex represents the interlayer exchange constant, which depends strongly on the thickness of the spacer layer.

To derive the equation of motion for the skyrmion, we start with the Landau-Lifshitz-Gilbert (LLG) equation, incorporating both field-like and damping-like spin-orbit torques. The LLG equation for each layer, i, is given as follows:

$$\frac{\partial \boldsymbol{m}_i}{\partial t} = -\gamma \mathbf{m}_i \times \mathbf{H}_{\mathrm{eff},i} + \alpha \mathbf{m}_i \times \frac{\partial \mathbf{m}_i}{\partial t} + \mathbf{T}_{\mathrm{SOT},i}, \quad \text{(S4)}$$

where γ is the gyromagnetic ratio, α is the phenomenological Gilbert damping parameter, $\mathbf{H}_{\mathrm{eff},i}$ is the effective magnetic field and $\mathbf{T}_{\mathrm{SOT},i}$ is the spin-orbit torque (SOT) of layer i, respectively. The field-like and damping-like SOT are given as follows:

$$\mathbf{T}_{\mathrm{SOT},i} = \tau_{\mathrm{DL}}\mathbf{m}_i \times [\mathbf{m}_i \times (\hat{z} \times \mathbf{j})] + \tau_{\mathrm{FL}}\mathbf{m}_i \times (\hat{z} \times \mathbf{j}). \qquad \text{(S5)}$$

By assuming that the skyrmion tube spin texture in the multilayer remains rigid during its steady state motion, we can model the skyrmion dynamics in each layer using the Thiele equation:

$$\mathcal{G}_i \times \dot{\mathbf{R}}_i - \alpha\mathcal{D}_i\dot{\mathbf{R}}_i - \tau_{\mathrm{FL}}\mathbf{F}_{\mathrm{FL},i} - \tau_{\mathrm{DL}}\mathbf{F}_{\mathrm{DL},i} = 0, \qquad \text{(S6)}$$

where $\mathcal{G}_i$ is the gyrotropic tensor producing the gyrotropic force perpendicular to the current, $\mathcal{D}_i$ is the dissipative tensor describing the generalized drag force acting along the current and, $\mathbf{F}_{\mathrm{FL},i}$ and $\mathbf{F}_{\mathrm{DL},i}$ are the effective forces arising from the field-like and damping-like components of SOT respectively. These quantities are defined as follows

$$\mathcal{G}_i|_{p,q} = \int_S d^2\mathbf{r}\left[\mathbf{m}_i \cdot \left(\partial_p\mathbf{m}_i \times \partial_q\mathbf{m}_i\right)\right], \qquad \text{(S7)}$$

$$\mathcal{D}_i|_{p,q} = \int_S d^2\mathbf{r}\left(\partial_p\mathbf{m}_i \cdot \partial_q\mathbf{m}_i\right), \qquad \text{(S8)}$$

$$F_{\mathrm{FL},i}|_p = \int_S d^2\mathbf{r}(\hat{z} \times \mathbf{j}) \cdot \partial_p\mathbf{m}_i, \qquad \text{(S9)}$$

$$F_{\mathrm{DL},i}|_p = \int_S d^2\mathbf{r}\left[(\hat{z} \times \mathbf{j}) \times \partial_p\mathbf{m}_i\right] \cdot \mathbf{m}_i. \qquad \text{(S10)}$$

Upon solving the Thiele equations for the skyrmion velocity $\left(\dot{R}_{x.i}, \dot{R}_{y,i}\right)$, we can calculate the skyrmion Hall angle, $\vartheta_{\mathrm{SkHE}}$, which is given by,

$$\tan\vartheta_{\mathrm{SkHE},i} = -\frac{G(\tau_{\mathrm{FL}}F_{\mathrm{FL},x}+\tau_{\mathrm{DL}}F_{\mathrm{DL},x})+\alpha\mathcal{D}_{xy}(\tau_{\mathrm{FL}}F_{\mathrm{FL},x}+\tau_{\mathrm{DL}}F_{\mathrm{DL},x})-\alpha\mathcal{D}_{xx}(\tau_{\mathrm{FL}}F_{\mathrm{FL},y}+\tau_{\mathrm{DL}}F_{\mathrm{DL},y})}{G(\tau_{\mathrm{FL}}F_{\mathrm{FL},y}+\tau_{\mathrm{DL}}F_{\mathrm{DL},y})+\alpha\mathcal{D}_{yy}(\tau_{\mathrm{FL}}F_{\mathrm{FL},x}+\tau_{\mathrm{DL}}F_{\mathrm{DL},x})-\alpha\mathcal{D}_{xy}(\tau_{\mathrm{FL}}F_{\mathrm{FL},y}+\tau_{\mathrm{DL}}F_{\mathrm{DL},y})}. \qquad \text{(S11)}$$

The index "i" for the tensor elements is omitted here for the sake of brevity. We can further simplify the expression for $\vartheta_{\mathrm{SkHE}}$ in Eq. (S11) by assuming that the skyrmion structure in the multilayer stack is a centrosymmetric cylindrical tube, which allows us to write the magnetization in each layer as being described by both the radius r and the azimuthal angle ϕ,

i.e., $\mathbf{m}_i(r,\phi) = [\sin\Theta(r)\cos\Phi(\phi), \sin\Theta(r)\sin\Phi(\phi), \cos\Theta(r)]$. This yields the following expression for $\vartheta_{\mathrm{SkHE}}$:

$$\tan\vartheta_{\mathrm{SkHE},i} = \frac{\alpha\mathcal{D}_{xx}\sin(\eta-\nu)-(\alpha\mathcal{D}_{xy}+4\pi)\cos(\eta-\nu)}{(4\pi-\alpha\mathcal{D}_{xy})\sin(\eta-\nu)+\alpha\mathcal{D}_{yy}\cos(\eta-\nu)}, \quad \text{(S12)}$$

where the current is injected into each layer at an angle η with respect to the x axis. It is important to note here that we have assumed the profile function $\Theta(r)$ of the skyrmion to vary from 0 (or π) at $r = 0$ to π (or 0) as $r \to \infty$. Equation (S12) reproduces well the SkHE measurements at low current densities (namely the low velocity regime). However, at higher current densities (in the extreme case[11]), the skyrmion helicity changes over time requiring an additional degree of freedom (helicity) to be included in the equations of motion to account for these changes during the current-driven motion. This would introduce an additional force term in the Thiele equation, providing a more accurate depiction of the skyrmion dynamics. However, this is beyond the scope of the current work. Instead, we calculate the accurate Hall angles by means of micromagnetic simulations, which corroborate the experimental observations.

Note 5
Micromagnetic modelling

In our simulations, the skyrmion in each layer was initialized with opposite polarization, and the energy was minimized to obtain a stable skyrmion tube configuration. The skyrmions were then driven using spin-orbit torques (SOTs) and currents, and the magnetization state was recorded at intervals of 100 picoseconds. We systematically varied the current strengths from 1×10^{11} A m^{-2} to 10^{12} A m^{-2} while fixing the spin Hall angle to be 0.1 (as obtained from the ST-FMR measurements in supplementary Note 3). For each current strength, we tracked the skyrmion positions and averaged the skyrmion Hall angle across all layers. Furthermore, the Thiele tensors (Eqs. S7-S12) were calculated for both directions of the applied current, and their difference is shown in supplementary Fig. 5c-d.

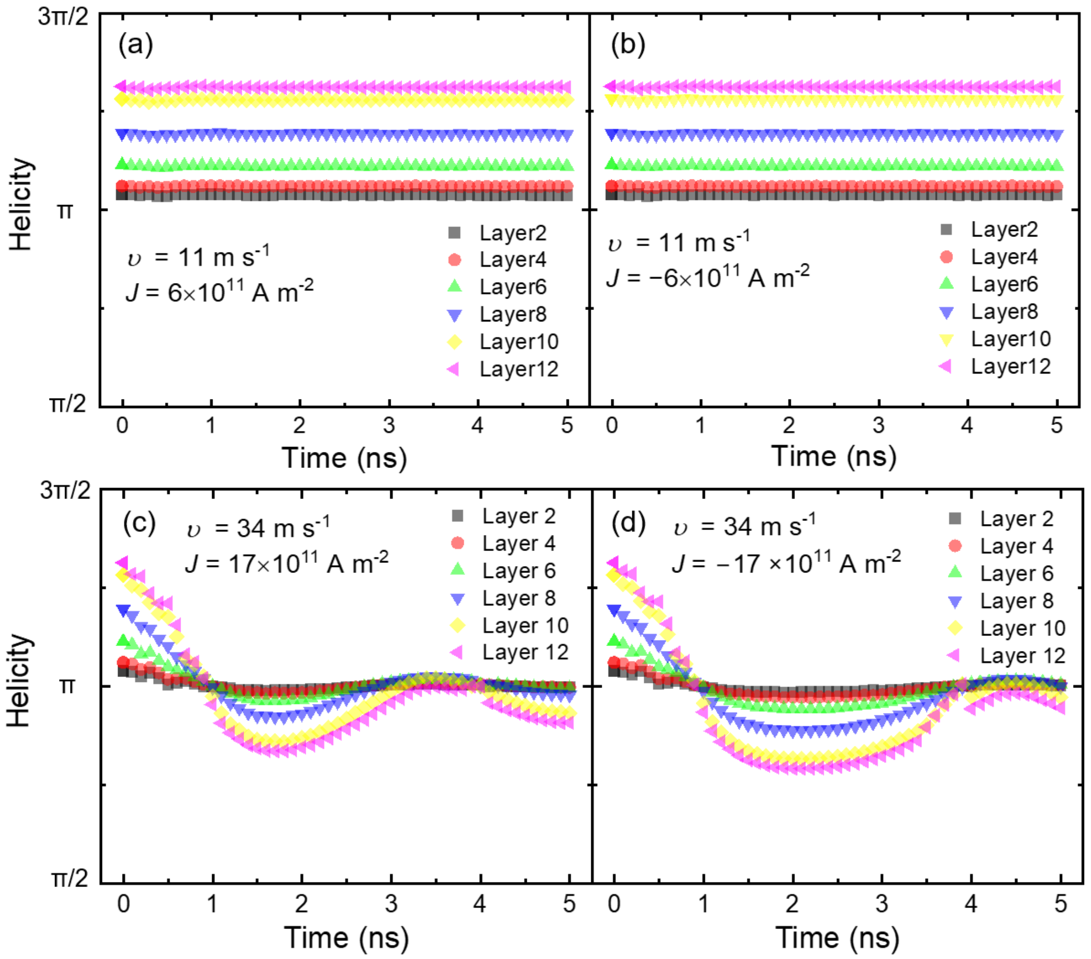

Supplementary Figure 5 | Helicity changes of hybrid skyrmions under different dynamic regimes. **a,** Helicity of every alternating layer during a 5 ns pulse width under a positive pulse direction ($J = 6\times10^{11}$ A m^{-2}) in region 1. **b,** Helicity of every alternate layer during a 5 ns pulse width under a negative pulse direction ($J = -6\times10^{11}$ A m^{-2}) in region 1. **c,** Helicity of every alternate layer during a 5 ns pulse width under a positive pulse direction ($J = 17\times10^{11}$ A m^{-2}) in region 2. **d,** Helicity of every alternate layer during a 5 ns pulse width under a negative pulse direction ($J = -17\times10^{11}$ A m^{-2}) in region 2. The helicity transitions from hybrid type to Néel type during the pulse duration in region 2.

Note 6

Systematic micromagnetic simulation:
magnetic compensation dependence of the static and dynamic properties

Here, we investigate how the magnetic compensation affects both the helicity distribution through the film thickness and the current-driven skyrmion dynamics, thereby elucidating the underlying physics. Supplementary Figure 6a plots the equilibrium helicity in the topmost and bottommost layers of antiferromagnetically coupled skyrmion tubes as a function of compensation. At 80 % and 60 % compensation, the helicity is uniform across all layers, corresponding to a pure Néel-type configuration. At 50 % compensation, a helicity gradient arises, yielding a hybrid Néel-Bloch character. As compensation falls to 40 %, the Bloch component grows more pronounced, and at 20 %, the domain-wall texture approaches a nearly pure Bloch-type, as expected.

Supplementary Figures 6b-e show the corresponding current-induced dynamics. At 20 % compensation, a pronounced non-reciprocal skyrmion Hall effect appears due to the large helicity difference between the top and bottom layers. As compensation increases, this non-reciprocal response is observed only at higher current densities. At 60 % and 80 % compensation, the skyrmion Hall angle is symmetric with respect to current polarity, consistent with a pure Néel-type structure lacking Bloch components, and a non-reciprocal effect becomes insignificant.

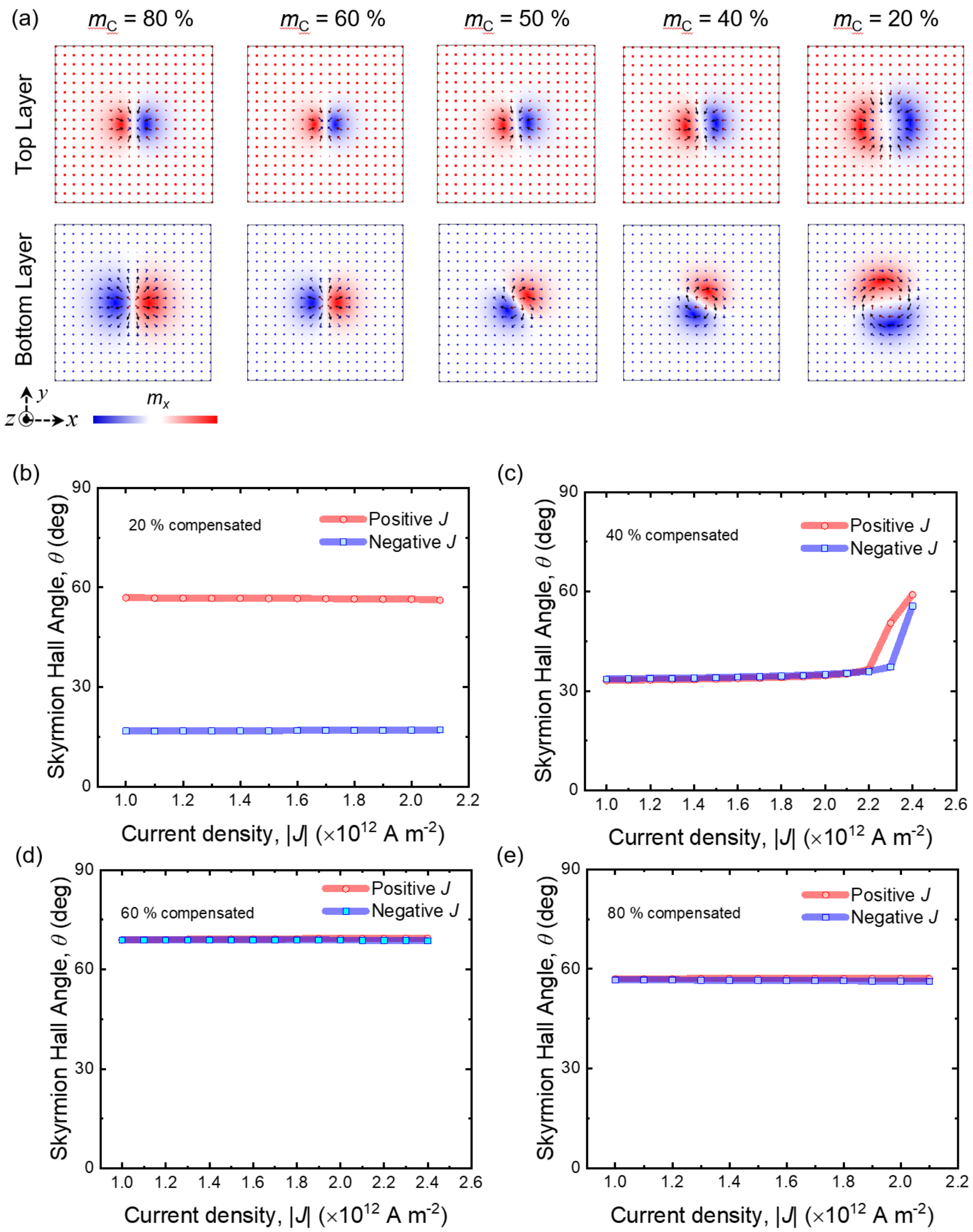

Supplementary Figure 6 | Systematic micromagnetic simulations of antiferromagnetic hybrid chiral skyrmion tubes. **a**, 2D magnetization profiles of the topmost and bottommost layers for skyrmions in SyAFM stacks with 80 %, 60 %, 50 %, 40 %, and 20 % magnetic compensation, respectively. Arrow directions indicate the magnetization orientation within each layer, while color variations denote the magnetization direction along the *x*-axis. The skyrmion Hall angle as a function of current density for varying degrees of magnetic compensation: **b**, 20%, **c**, 40%, **d**, 60%, and **e**, 80%.

Supplementary References

1 Zhang, X., Zhou, Y. & Ezawa, M. Magnetic bilayer-skyrmions without skyrmion Hall effect. *Nature Communications* **7**, 10293 (2016). https://doi.org/10.1038/ncomms10293

2 Koshibae, W. & Nagaosa, N. Theory of skyrmions in bilayer systems. *Scientific Reports* **7**, 42645 (2017). https://doi.org/10.1038/srep42645

3 Dohi, T., Duttagupta, S., Fukami, S. & Ohno, H. Formation and current-induced motion of synthetic antiferromagnetic skyrmion bubbles. *Nature Communications* **10**, 5153 (2019). https://doi.org/10.1038/s41467-019-13182-6

4 Benitez, M. J. *et al.* Magnetic microscopy and topological stability of homochiral Néel domain walls in a Pt/Co/AlOx trilayer. *Nature Communications* **6**, 8957 (2015). https://doi.org/10.1038/ncomms9957

5 Fallon, K. *et al.* Quantitative imaging of hybrid chiral spin textures in magnetic multilayer systems by Lorentz microscopy. *Physical Review B* **100**, 214431 (2019). https://doi.org/10.1103/physrevb.100.214431

6 Denneulin, T. *et al.* Visibility and Apparent Size of Néel-Type Magnetic Skyrmions in Fresnel Defocus Images of Multilayer Films. *Microscopy and Microanalysis* **27**, 1356-1365 (2021). https://doi.org/10.1017/s1431927621012927

7 Sud, A. *et al.* Tunable magnon-magnon coupling in synthetic antiferromagnets. *Physical Review B* **102** (2020). https://doi.org/10.1103/physrevb.102.100403

8 Sud, A., Yamamoto, K., Suzuki, K. Z., Mizukami, S. & Kurebayashi, H. Magnon-magnon coupling in synthetic ferrimagnets. *Physical Review B* **108**, 104407 (2023). https://doi.org/10.1103/physrevb.108.104407

9 Rogdakis, K. *et al.* Spin transport parameters of NbN thin films characterized by spin pumping experiments. *Physical Review Materials* **3**, 014406 (2019). https://doi.org/10.1103/physrevmaterials.3.014406

10 Sud, A. *et al.* Parity-controlled spin-wave excitations in synthetic antiferromagnets. *Applied Physics Letters* **118**, 032403 (2021). https://doi.org/10.1063/5.0037427

11 Back, C. *et al.* The 2020 skyrmionics roadmap. *Journal of Physics D: Applied Physics* **53**, 363001 (2020). https://doi.org/10.1088/1361-6463/ab8418
