# Supplementary material for: Observation of a non-reciprocal skyrmion Hall effect of hybrid chiral skyrmion tubes in synthetic antiferromagnetic multilayers
Source: Nat Commun. 2025 Sep 26;16:8285. doi: 10.1038/s41467-025-63759-7 (PMC12474975; doi:10.1038/s41467-025-63759-7)
Supplement: Supplementary file 3 — Description of Additional Supplementary Files [file 41467_2025_63759_MOESM3_ESM.pdf]

Description of Additional Supplementary Files

Supplementary Movie 1: Tracking SyAFM skyrmion tube motion J = +13x10^{11} A m^{-2}, Pulse width 5 ns.

Supplementary Movie 2: Tracking SyAFM skyrmion tube motion J = -13x10^{11} A m^{-2}, Pulse width 5 ns.

Supplementary Movie 3: Current-induced SyAFM hybrid chiral skyrmion tubes in region 1, positive current pulse.

Supplementary Movie 4: Current-induced SyAFM hybrid chiral skyrmion tubes in region 1, negative current pulse.

Supplementary Movie 5: Current-induced SyAFM hybrid chiral skyrmion tubes in region 2, positive current pulse.

Supplementary Movie 6: Current-induced SyAFM hybrid chiral skyrmion tubes in region 2, negative current pulse.
